# Efficacy and safety of a combined treatment of sodium stibogluconate at 20mg/kg/day with upper maximum daily dose limit of 850mg and Paromomycin 15mg/kg/day in HIV negative visceral leishmaniasis patients. A retrospective study, northwest Ethiopia

**DOI:** 10.1371/journal.pntd.0009713

**Published:** 2021-08-31

**Authors:** Aschalew Tamiru, Rezika Mohammed, Saba Atnafu, Girmay Medhin, Asrat Hailu

**Affiliations:** 1 Leishmaniasis Research and Treatment Center, University of Gondar, College of Medicine and Health Science, Gondar, Ethiopia; 2 Department of Internal Medicine, University of Gondar, College of Medicine and Health Sciences, Gondar, Ethiopia; 3 Leishmaniasis Research and Treatment Center, University of Gondar, College of Medicine and Health Science, Gondar, Ethiopia; 4 Aklilu Lemma Institute of Pathobiology, Addis Ababa University, Addis Ababa, Ethiopia; 5 Department of Microbiology, Immunology and Parasitology, College of Health Sciences, Addis Ababa University, Addis Ababa, Ethiopia; University of Texas Medical Branch, UNITED STATES

## Abstract

**Background:**

Visceral leishmaniasis (VL) is one of the most neglected tropical infectious diseases. It is fatal if left untreated. The objective of this study was to assess the efficacy and safety of 17-day injections of combined regimen of sodium stibogluconate and paromomycin (SSG/PM) in HIV-negative VL patients.

**Methods:**

A retrospective analysis of medical records of VL patients treated in the University of Gondar Hospital during period 2012–2019 was carried out.

**Results:**

A total of 2836 patients were treated for VL from 2012 to 2019. Of these 1233 were treated with SSG-PM, and 1000 of them were included in the study. Initial cure was achieved in 922 (92.2%) patients. The frequency of treatment failure, treatment interruptions, default and deaths respectively were 30 (3%), 20 (2%), 13 (1.3%) and 15 (1.5%). Among 280 patients who completed 6-month follow up, the final cure was 93.9% (263/280), 4 (1.4%) relapsed and 13 (4.6%) developed post-kala-azar dermal leishmaniasis (PKDL). The most common adverse events (AEs) were raised liver transaminases (35.1%; 351 patients), injection site pain (29.1%, 291 patients) and raised serum alpha-amylase (29.1%, 291 patients). Factors associated with poor treatment outcomes were sepsis, pneumonia, and adverse events.

**Conclusion:**

A combination of SSG at 20mg/kg with upper daily maximum dose of 850mg and PM was effective for achieving initial cure at end of treatment and safe for treatment of HIV negative VL patients in northwestern Ethiopia. Our data are consistent with previous reports and confirms effectiveness of SSG/PM treatment regimen in the Eastern African countries. Efficacy at 6-months (93.9%) was estimated on data derived from patients who completed follow up and needs to be interrogated by future studies.

## Author summary

Visceral leishmaniasis is one of the most neglected tropical diseases caused by the parasite of the genus leishmania. We assessed the efficacy and safety of a 17-day combined regimen of sodium stibogluconate 20mg/kg /day with daily upper maximum dose limit of 850mg and paromomycin 15mg/kg/day injections in HIV negative visceral leishmaniasis patients. We also identified factors associated with poor treatment outcomes in HIV negative VL patients treated with SSG/PM. Seventy two percent of the study participants developed at least one AE during the initial treatment course but most AEs were reversible. The overall initial efficacy was 92.2% which is comparable with previous study results in East Africa. Among patients who completed six-month follow-up, the efficacy was 93.9%, which appears to be biased due to the small proportion of patients (29%) evaluated at 6-month follow-up. The results affirm that the combination of sodium stibogluconate (20mg/kg b.wt) and paromomycin (15mg/kg b.wt) given for 17 days is still effective in Eastern Africa VL endemic countries. This data applies to VL patients who are HIV negative and not presenting with severe signs/symptoms.

## Introduction

Visceral leishmaniasis (VL) is among the most neglected tropical diseases caused by the protozoan parasite of the genus Leishmania (*Leishmania donovani* and *Leishmania infantum* [1]. Its transmission occurs through the bite of phlebotomine sand flies although other routes of transmission including blood transfusion, intravenous drug use, organ transplantation, and congenital and laboratory accidents are also implicated [2]. In East Africa and Indian sub-continent where *L*. *donavani* is the cause of visceral leishmaniasis, transmission is anthroponotic (human to human). In the Mediterranean basin and Latin America where *L*. *infantum* infection is common, and transmission is zoonotic[1]. The typical clinical features of VL includes fever, weight loss, loss of appetite, splenomegaly, weakness, leucopenia, thrombocytopenia, and anemia. Globally, the annual incidence of VL is approximately 0.2 to 0.4 million. Over 90% of global VL cases occur in six countries: India, Bangladesh, Sudan, South Sudan, Ethiopia and Brazil [3].

The first report of VL cases in Ethiopia was in the Omo River valley in 1942 [4]. Since then, it has been spreading and imposing serious public health threats. It commonly occurs in arid and semi-arid lowlands [5]. Of the endemic areas, the northwest region particularly the Metema-Humera foci accounts for 90% of VL case burden according to data from KalaCore Ethiopia. In Ethiopia, its incidence is estimated to be between 3700–7400 cases per year [4]. The disease has rapidly spread to highland areas as exemplified by epidemics of VL in 2005 in the district of Libo Kemkem in Amhara Regional State where the first epidemics of VL occurred [6].

Pentavalent antimonials [sodium stibogluconate and meglumine antimoniate] have been the cornerstone of VL treatment except where it was abandoned [7,8]. Currently, a combination of SSG at 20mg/kg/day intravenously or intramuscularly and PM at 15mg/kg/day intramuscularly is the first-line treatment for HIV negative VL patients in Eastern Africa including Yemen. Liposomal amphotericin B (Lamp-B) is used as the second-line treatment owing to its wide safety margin [5]. In the Ethiopian treatment guidelines, second-line treatments are indicated for patients with HIV co-infection, treatment failure, severe VL, severe anemia, severe malnutrition, toxicity, relapse, pregnancy and extremes of age (below 2 or above 50 years) [9].

In Eastern African countries where the causative agent for VL (*L*. *donovani)* is highly virulent, drug unresponsiveness had been reported [10–12]. In a multicenter non-inferiority clinical trial conducted in East Africa comparing three regimens of VL drugs (SSG/PM, SSG, and PM), efficacy of PM injections of 21 days was generally low in Ethiopia [13]. Given the low efficacy of PM, there is no guarantee that *L*. *donovani* parasite in the region continues to be sensitive to the current drugs and needs continuous assessment.

The SSG/PM combination treatment was first evaluated in a clinical setting in Kenya in 1990 [14]. A retrospective cohort study in southern Sudan reported that the initial cure rate among patients treated with SSG/PM was 97% compared with 92.4% among patients treated with SSG monotherapy[15]. In a randomized comparative trial in Eastern Africa (Sudan, Kenya and Ethiopia), the efficacy of SSG & PM was comparable to SSG (91.4% versus 93.9%, difference = 2.5%, 95% CI: -1.3 to 6.3%) [16]. Another observational study in Eastern Sudan reported an initial and final efficacy of 93% and 86%, respectively [17]. Currently, 17 injections of a combination of SSG at 20mg/kg/day without upper maximum daily dose limit (850mg) and PM at 15mg/kg/day is extensively used in East Africa as first-line drug following the 2010 World Health Organization (WHO) recommendation [5]. Accordingly, the VL treatment center at the University of Gondar Hospital [i.e., the Leishmaniasis Research and Treatment Center (LRTC)] has been using the WHO recommended 17-day SSG/PM combination treatment but holding on to the 850mg upper maximum daily dose. The objective of this study was to assess the efficacy and safety of SSG/PM when used in a routine setting and to identify factors associated with poor treatment outcomes.

## Materials and methods

### Ethics statement

The study was approved by Scientific and Ethics Review Committee (SERC) of Center for Innovative Drug Development and Therapeutics Trials for Africa (CDT-Africa)-Addis Ababa University-College of Health Sciences and University of Gondar Institutional Review Board (IRB). The confidentiality of patients’ medical record was maintained throughout. As the study involved a retrospective patient card review, informed consent from VL patients was impossible to obtain. To ensure anonymity, patients’ medical registration number and codes were used to identify medical records. Patient unique identifiers of individual subjects (e.g., name, address, phone number, etc.) were not captured during data extraction.

### Study setting/Context

Gondar Leishmaniasis Research and Treatment Center (LRTC) is located in Northwest Ethiopia at a distance of 738km from the capital city, Addis Ababa. The Drugs for Neglected Disease initiative (DNDi) in collaboration with the University of Gondar Hospital established the LRTC in May 2005. Since then, the treatment center has been conducting various clinical trials in search of safe and efficacious treatment for VL. Moreover, the center has been serving the community by providing leishmaniasis diagnostic and treatment services. The center has a ward with 24 beds, a separate laboratory, and a pharmacy. The health professionals are trained on Good Clinical Practice (GCP) to provide quality-assured standard care to VL patients and also to involve in clinical trials when needed.

The center also provides HIV testing and counseling services. HIV tests are carried out based on the national HIV counseling and testing algorithm. All VL patients routinely undergo HIV counseling and testing as stipulated in the national guideline.

### Study design

A retrospective review of medical records of VL patients treated with SSG/PM in the period between January 1, 2012 to June 30, 2019 was carried out. Data was extracted from medical records of VL patients; specifically medical records of VL patients admitted to the University of Gondar Hospital Leishmaniasis Research and Treatment Center (UoGH LRTC).

### Study participants

The age of study participants ranged from 4 to 60 years. VL diagnosis was confirmed parasitologically or clinically and/or serologically at UoGH LRTC from January 1, 2012 to June 30, 2019. HIV negative VL patients were treated with SSG/PM following the national guidelines. Patients with VL relapse, incomplete medical records (i.e., a chart with no record available for safety and outcome assessment), and HIV positives were excluded from the study. According to the national treatment guideline, patients with concomitant conditions including abnormal liver/kidney injury and other complications are not treated with SSG/PM. However, if any patient with such conditions was treated with SSG/PM, the data was still captured.

Patients coming to the treatment center were either self-presenting or referred from other health facilities. VL suspected patients were evaluated by well-experienced health professionals.

### Diagnosis

VL diagnoses were carried out based on Ethiopian guidelines [9]. Individuals who fulfilled the VL clinical case definition of WHO (fever of greater than 2 weeks, splenomegaly and/or lymphadenopathy, or either loss of weight, anemia, or leucopenia) were further tested by rk-39 rapid diagnostic test and subsequently by parasitological procedures in splenic and bone marrow aspirates. Patients were eligible for treatment if aspiration results turned microscopy positive in Giemsa-stained specimens. Patients whose aspiration result became negative but fulfilled the clinical case definition (with or without a positive serological test for leishmania) were still treated.

### Treatment protocols

VL patients without VL complications and concomitant diseases (clinically significant edema, severe disease, organ (liver, kidney and hear) failure, malnutrition and HIV) received a combination of SSG at 20mg/kg/day IM/IV at upper maximum daily dose of 850mg and paromomycin sulphate 15mg/kg (11mg PM base) for 17 days. Our review of the patient charts show that at the end of treatment (day 17), initial treatment outcome was evaluated either by performing a test of cure (microscopic examination of the parasite in spleen or bone marrow aspirate) or by clinical evaluation.

VL patients who either became parasite positive and/or judged to have no clinical improvement were either given additional 13 injections of SSG alone to add up to 30 doses or shifted to the second-line treatment (Liposomal amphotericin B at 5mg/kg /day, 6 doses) based on clinician decision.

Weekly laboratory investigations (hematology and biochemical tests to monitor liver and renal functions) were performed. Clinical and laboratory profiles were documented until treatment completion. For patients who developed adverse events, drugs were discontinued either temporarily or permanently based on AE severity. Patients were appointed to come back to the treatment center (mandatory appointment) for final assessment of outcomes 6 months after end of treatment. Patients were also instructed to report back 3 months after treatment completion in case they noticed re-emergence of VL symptoms.

### Data collection and management

Data collection was carried out by trained clinical personnel who had the experience of working in visceral leishmaniasis clinics or health professional oriented to clinical aspects of the disease. A patient chart was used to demonstrate how to review and collect data. A standard data collection format was used to address the objectives described above and included variables of the following categories: socio-demographic characteristics such as age, gender, and migration status; laboratory characteristics that encompass hematologic profiles such as white blood cell count, hemoglobin, and platelet count; biochemical assessments such as alanine aminotransferase (ALT), and aspartate aminotransferase (AST) for liver function tests; blood urea nitrogen (BUN) and creatinine for renal function test, and alpha amylase for pancreatic function test; clinical characteristics, which include fever, weight loss, loss of appetite, splenomegaly, hepatomegaly, bleeding, lymphadenopathy, and cough; concomitant diseases (pneumonia, tuberculosis (TB), sepsis, diarrheal disease, otitis media, malaria and others). Participants’ body weight and height were also available in the records and captured in the data base.

Data entry in the Case Record Forms was checked for quality on a daily basis. Data entry was verified against the patients’ medical records with corrections made accordingly. Data was entered in Epi-data version 3.1.

### Data analysis

The data were cleaned and exported to SPSS software version 20 for statistical analysis. Chi-square test and Fischer Exact test were used to compare proportions of categorical variables. A bivariate logistic regression was used to identify factors associated with poor treatment outcomes. Multivariable logistic regression model was used to adjust for potential confounding effects and to generate adjusted measures of effect. P-value less than 0.05 was used to report statistical significance. A 95% CI that included “1” indicated statistical non-significance.

### The following definitions were adapted

**Initial cure:** clinical cure or negative aspiration result at the end of treatment.

**Definitive cure:** clinical cure or negative aspiration result at six months.

**Clinical cure:** the absence of clinical signs and symptoms and improvement in hematological profiles either at the end of treatment or 6 months after VL treatment.

T**reatment failure:** presence of signs and symptoms of VL and/or parasite in the spleen or bone marrow aspirate at the end of treatment and up to one-month post end of treatment.

**Defaulter:** a patient who took less than 14 doses of SSG/PM due to the patient leaving the hospital.

**Treatment interruptions due to drug toxicity:** discontinuation of drug due to drug toxicity.

**Poor treatment outcome:** treatment failures, defaulters, deaths, and treatment interruptions due to drug toxicity.

**Adverse event:** abnormal biochemical laboratory results from the baseline and a self-reported patient complaint related to drugs.

**Clinical pancreatitis:** the presence of signs and symptoms of pancreatitis and raised amylase which leads to treatment interruptions.

N**on-clinical pancreatitis:** raised amylase with no clinical signs and symptoms of clinical pancreatitis.

## Results

### Patient characteristics

A total of 2836 VL patients were treated in UoGH LRTC between January 1, 2012, and June 30, 2019. Nearly 57% of VL patients (n = 1603) who received drugs other than SSG/PM (AmBisome, SSG monotherapy and AmBisome and Miltefosine combinations) were excluded. Furthermore, among 1233 patients treated with SSG/PM, 233 patients were excluded for reasons such as HIV co-infection, lost charts, and incomplete medical record (S1 Fig). Eventually data was extracted from records of 1000 study participants and included in the analysis. The majority (n = 834, 83.4%) of participants were adults with age 18 years and above. The rest 166 patients (16.6%) were age below 18 years. The median [interquartile range (IQR)] age of the study participants was 23 (20–27), and 996 (99.6%) were male VL patients. Eighty-five percent of patients were seasonal migrant workers, while the rest (15%) were residents of VL endemic areas (Table 1).

**Table 1:** Baseline Clinical Characteristics of VL patients Treated with SSG/PM at UoGH-LRTC from January 1, 2012 to June 30, 2019 Northwest Ethiopia (N = 1000).

Characteristics	Number	Percent
Fever	995	99.5
Splenomegaly	964	96.4
Weight loss	945	94.5
Loss of appetite	928	92.8
Cough	485	48.5
Hepatomegaly	289	28.9
Edema	279	27.9
Bleeding	238	23.8
Jaundice	130	13
Lymphadenopathy	20	2
Spleen size at admission [Mean (±SD)] in cm	-	9.4 (4.5)
Method of diagnosis		
Parasitological	874	87.4
Clinical and/or serological	126	126 (12.6)

The median (IQR) of the duration of illness before admission was 4 (3–8) weeks. On admission, 995(99.5%) patients had fever, 95% had a history of weight loss and 93% had loss of appetite The median (and range) of weight was 24 (19–58) kgs in patients with age ≥ 18 years of age. Similarly, the median (and range) of weight was 23 (5–58) kgs in patient group with weight > 42.5kg.

The median (and IQR) of white blood cell count, hemoglobin, and platelet at admission were, 1800 (1300–2500) cells/μl, 8.5 (7–9.9) mg/dl and 68,000 (44,000–105,000) cells/μl, respectively. With regards to baseline biochemical tests, the median (IQR) of alanine transaminase (ALT), aspartate transaminase (AST) and alpha amylase were 39(23–36), 76.2 (44–134), 177.5 (124.8–254.3) Unit/L, respectively. The median (IQR) of blood urea nitrogen (BUN), and creatinine were 11.4 (8.9–14.6) mg/dl, respectively.

### Efficacy

Of the total 1000 VL patients treated with SSG and PM, initial cure was achieved in 922 (92.2%). (**Table** 2).

**Table 2:** Overall initial treatment outcomes of VL patients treated with SSG and PM combination from January 1, 2012, to June 30, 2019 (N = 1000).

Initial treatment outcome	Number	Percent
Cured	922	92.2
Failed	30	3
Treatment interruptions due to drug toxicity	20	2
Defaulted	13	1.3
Died	15	1.5

In the analysis of initial treatment outcomes of VL patients stratified by patients’ body weight at admission (≤ 42.5kg and > 42.5kg), there was no statistically significant difference in the efficacy (90.4% Vs 92.7%) of SSG/PM between the groups (P = 0.27).

Overall, 972 patients were appointed to come for a six month follow up visit, but only 280 (28.8%) adhered. The remaining 692 (71.2%) were lost follow-up. Among the 280 patients who attended six months follow up visit, cure was achieved in 263 (93.9%). Similarly, relapse and PKDL were noted in 4(1.4%) and 13 (4.6%) patients respectively. In the worst-case scenario, i.e., considering all lost follow-up as relapse, PKDL and death, definitive cure rate would be 27.1% (263 cured among 972 treated patients) and PKDL and relapse respectively would be 1.3% (13 patients) and 0.4% (4 patients). The subgroup analysis by residency status also showed no statistically significant difference was noted in the efficacy of SSG-PM at six month between resident and migrant patients (25.34%; 37/146 versus 27.4%; 226/826; p-value = 0.61).

### Safety

During the initial treatment period, AEs were found in 725 (72.5%) patients. The most common AEs were raised transaminase in 351 (35.1%), injection site pain in 291 patients (29.1%) and raised serum amylase in 291 (29.1%) (Table 3).

**Table 3:** Adverse events of VL patients treated with SSG/PM from January 1, 2012, to June 30, 2019, at UoGH-LRTC, Northwest Ethiopia (N = 1000).

Adverse events	N = 1000`n (%)
Vomiting	
Yes	37 (3.7)
No	963 (96.3)
Nausea	
Yes	8 (0.8)
No	992 (99.2)
Arthralgia	
Yes	3 (0.3)
No	997 (99.7)
Injection site pain	
Yes	291 (29.1)
No	709 (70.9)
Increased serum Transaminases	
Yes	351 (35.7)
No	649 (64.9)
Clinical Pancreatitis	
Yes	21 (2.1)
No	979 (97.9)
Increased serum creatinine	
Yes	88 (8.8)
No	912 (91.2)
Increased serum Amylase	
Yes	291 (29.1)
No	709 (70.9)
Cardiac arrhythmia	
Yes	6 (0.6)
No	994(99.4)
Thrombophlebitis	
Yes	3 (0.3)
No	997 (99.7)
Dyspepsia	
Yes	17 (1.7)
No	983 (98.3)
Hearing loss	
Yes	9 (0.9)
No	991 (99.1)

### Associated factors for poor treatment outcomes

The proportion of poor treatment outcomes of VL patients treated with a combination of SSG and PM was 78 (7.8%). Of, the variables fitted into the multivariate logistic regression model, sepsis, pneumonia and adverse events were significantly and independently associated with poor treatment outcomes.

VL patients with sepsis at admission had nearly 13 times increased odds of having poor treatment outcomes than those without sepsis (OR: 13.29, 95% CI; 3.57–49.57). Participants who experienced adverse events had 2.56 times increased risk of having poor treatment outcomes as compared to those without adverse event (OR: 2.56, 95% CI; 1.31–5.01). Participants who had pneumonia at admission were 1.7 times more likely to have poor treatment outcomes than those without the condition (OR: 1.73, 95% CI; 1.00–2.97) (Table 4).

**Table 4:** Associations of selected variables and Poor Treatment Outcomes (N = 1000).

Variables	Poor treatment outcome		Crude Odds ratio (COR) and 95% Confidence interval	Adjusted odds ratio (AOR) and 95% CI
	yes	No		
Edema				
Yes	31	248	1.32 (0.81–2.16)	1.35 (0.82–2.25)
No	47	674	1	1
Diarrhea				
Yes	1	59	0.19 (0.03–1.39)	0.14(0.17–1.16)
No	77	863	1	1
Pneumonia				
Yes	21	162	1.7 (1.02–2.93)	1.73 (1–2.97)
No	57	760	1	1
Sepsis				
Yes	5	6	10.46 (3.12–35.08)	13.29(3.57–49.57)
No	73	916	1	1
Spleen size				
< 14cm	62	801	1	1
≥14cm	16	121	1.7 (0.96–3.05)	1.7 (0.96–3.15)
Admission weight				
≤ 42.5	21	184	1	1
> 42.5	57	738	1.5 (0.87–2.5)	0.71(0.41–1.24)
Adverse Event				
Yes	67	658	2.44(1.27–4.69)	2.56(1.31–5.01)
No	11	264	1	1

## Discussion

This is a retrospective study that used data from medical records of VL patients treated with a combination of SSG at 20mg/kg with the upper maximum daily iv/im dose of 850mg and PM at 15mg/kg/day IM for 17 days. The vast majority of the study participants were males. Over 83% of study participants were adults with age greater than 18years due to the fact that young males (adults) more than females migrate from the highland areas to the lowlands of VL endemic foci (Ethio-Sudan borders; Metema, Humera and Abdurafi) in search of work. Nearly 57% of VL patients were treated with drugs other than SSG/PM (AmBisome, SSG or AmBisome and Miltefosine combination). Paromomycin stockouts, high number of VL-HIV co-infected cases, and severely ill patients at admission were the main reasons for treatments other than SSG/PM.

The initial cure achieved in the overall efficacy analysis and sub-group analysis by weight category were consistent with previous reports in East Africa [16,17]. The difference in the cure rate (90.4% Vs 92.7%) between patients with weight ≤ 42.5kg and > 42.5kg was not statistically significant. Efficacy of SSG /PM at six month was affected by many lost follow-ups. In this regard two major reasons for lost follow up are suggested; i) most patients were economically poor and live in remotest areas with limited access to transportation; ii) in practice, most patients who come any time before 6 months do not attend the final visit. Limiting follow-up time point to six month only unless indicated in exceptional medical condition, may improve follow up visit.

Final cure observed in this study (93.9%), albeit in a small proportion of patients evaluated, was comparable with a similar study reported by Atia [17]. The efficacy result that excluded lost-to follow-up might overestimate the result and should be carefully interpreted. Considering that resident patients could have better follow up attendance, subgroup analysis by residency status was done to see if better efficacy could be achieved at 6 month, but no statistically significant difference was observed between the groups (resident and migrant patients).

The number of PKDL cases reported at six month in the current study was low compared to a similar study in Sudan where it is commonly reported [18]. PKDL patients had been reported to act as the best reservoir especially in patients with concurrent HIV co-infection with high parasitemia [19].

The use of SSG at the upper maximum daily dose of 850mg IM/IV has several advantages; decreased toxicity, reduced risk of drug resistance, decreased need for a high volume of SSG, and hence decreased injection site pain. Its combined use with PM had been reported to have an acceptable efficacy profile as reported in previous studies[13,16]. However, WHO still recommends its use at 20mg/kg disregarding the upper maximum dose limit in all VL endemic regions. Our finding shows that the use of SSG with the upper maximum daily dose limit can effectively be used in combination with PM in East African countries.

At least one adverse event occurred in 725 (72.5%) of VL patients. An increase in liver transaminase enzyme was the most common AE that was also reported in a previous study within the region [13]. The overlapping toxicity of both SSG and PM on the liver could explain the higher number of patients who had raised liver transaminases. Furthermore, the fact that some VL patients with elevated liver transaminases at baseline were treated with SSG/PM because of shortage of second-line drug (i.e., liposomal amphotericin B), seemed to be predisposing the patients to further increases in liver transaminases. Injection site pain was more common as reported in the results of similar studies[16,20]. Of note, this AE had been a common complaint by patients at the study area during treatment. The large volume of SSG injection (maximum, 8.5ml) coupled with a burning type of pain by PM could explain the high rate of injection site pain. Clinical pancreatitis, even though not very common, resulted in drug interruptions and a change of treatment. The frequency of raised amylase level with no sign and symptoms of clinical pancreatitis was relatively high. In our study, cardiac toxicity was not common (only 0.6%) and was comparable with a study conducted in South Sudan[17]. However, it resulted in either death or treatment interruptions. The hearing loss reported in this study was obtained from self-reported evidence and lacked confirmatory diagnostic procedures such as audiometric examinations. However, grossly our study shows similar finding with studies that reported evidence of hearing toxicity (i.e. audiometric shift) in SSG/PM treated patients[13].

Information related to factors associated with poor treatment outcomes is pivotal in the management of VL. The proportion of poor treatment outcome was 7.8% which is lower than the previous study result (12.1%) reported from Ethiopia [21]. The difference could be due to the inclusion of those VL patients treated with a 17-day regimen of SSG/PM only. Socio-demographic factors such as age had been reported as risk factors for death and poor treatment outcomes [22,23] which was not replicated in the current study. Co-morbidities (sepsis and pneumonia) were significantly associated with poor treatment outcomes (Table 4). A similar study reported bacterial infection as a risk factor for the outcome variable[24]. Contrary to studies from Uganda and Brazil that reported edema as a risk factor for death [24,25], our study did not show a significant association with the outcome variable. Even though it is common observation that VL patients with high parasite load end up with treatment failure, we could not undertake the analysis as some patients lacked results of parasite load.

Even though SSG/PM injections of 17 days showed decreased toxicity as compared to SSG injections of 30 days, it has the potential to induce AEs such as cardiac toxicity, pancreatitis, liver and kidney injury. Occurrence of adverse events showed significant association with poor treatment outcomes. A similar study reported AEs such as increase in liver enzymes as a prognostic factor for death in Brazil [23]. Hence, strict monitoring of patients for AEs during treatment is needed to detect early and manage accordingly.

In this study, the high rate of lost to follow-up at 6 months after treatment is not a surprise given that follow-up reporting was not compulsory in a routine health services settings. This has led to lack of confidence in the estimates of definitive cure rate at 6 months. Procedures required for AE monitoring such as electrocardiography (ECG) and audiometry were not performed. Outcomes in lost to follow-up cases (death, PKDL, relapse, or cure) are not known. While this study can give a good estimate of the initial cure under routine conditions, the results of the final cure could be biased by these limiting factors. Hence, a cohort study where patients can be traced actively for follow up is needed to obtain a good estimate of the final treatment outcome.

## Conclusion and recommendations

The initial and final cure rate after treatment of VL cases with a combination of SSG at 20mg/kg with an upper maximum daily dose limit of 850mg and PM at 15mg/kg were 92.2% and 93.9% respectively. Most AEs related to SSG/PM were reversible. Sepsis, adverse events, and pneumonia should be identified in the course of treatment so as to switch to second line anti-leishmanial treatments and to intervene with supportive treatments. The therapeutic use of SSG/PM combination in HIV negative VL patients and in patients without severe illness is expected to continue in northwestern Ethiopia and in Eastern African countries until better treatments are found. As the combination of SSG/PM is currently used in patients without co-morbid conditions, HIV co-infections and severe VL, this study illustrates the need for better treatments that are safe and effective for treating VL with a spectrum of clinical conditions is a high priority.

## Supporting information

S1 Box Reference laboratory test values. (DOCX)

S1 Fig Flow diagram showing eligibility of the study population. (TIF)

S1 Data Data file. (XLSX)
