# Supplementary material for: Efficacy and safety of a combined treatment of sodium stibogluconate at 20mg/kg/day with upper maximum daily dose limit of 850mg and Paromomycin 15mg/kg/day in HIV negative visceral leishmaniasis patients. A retrospective study, northwest Ethiopia
Source: PLoS Negl Trop Dis. 2021 Aug 31;15(8):e0009713. doi: 10.1371/journal.pntd.0009713 (PMC8437273; doi:10.1371/journal.pntd.0009713)
Supplement: S1 Box — (DOCX) [file pntd.0009713.s001.docx]

|  |  | **Lower Limit** | **Upper Limit** | **Unit** |
| --- | --- | --- | --- | --- |
| **Hematology** |  |  |  |  |
| White Blood Cell (WBC) x 10^3^ |  | 3.2 | 8.8 | Cells/ml |
| Hemoglobin | Male | 11.5 | 18 | g/dl |
|  | Female | 11 | 16.7 | g/dl |
| Platelet x 10^3^ |  | 128 | 432 | Cells/ml |
| **Clinical chemistry** |  |  |  |  |
| Aspartate Transaminase (AST) |  | 1 | 37 | Unit/Liter (U/L) |
| Alanin Transmainase (ALT) |  | 0 | 42 | U/L |
| Bilirubin total |  | 0 | 1.1 | mg/dl |
| Blood Urea Nitrogen (BUN) |  | 4.7 | 23.4 | mg/dl |
| Creatinine |  | 0.6 | 1.1 | mg/dl |
| Alpha-Amylase |  | 0 | 220 | U/L |

Reference laboratory test values

**Reference**

1. Reference laboratory test values. University of Gondar Leishmaniasis Research and Treatment Centre 2018.
