# Supplementary figures and images for: Efficacy and safety of a combined treatment of sodium stibogluconate at 20mg/kg/day with upper maximum daily dose limit of 850mg and Paromomycin 15mg/kg/day in HIV negative visceral leishmaniasis patients. A retrospective study, northwest Ethiopia
Source: PLoS Negl Trop Dis. 2021 Aug 31;15(8):e0009713. doi: 10.1371/journal.pntd.0009713 (PMC8437273; doi:10.1371/journal.pntd.0009713)

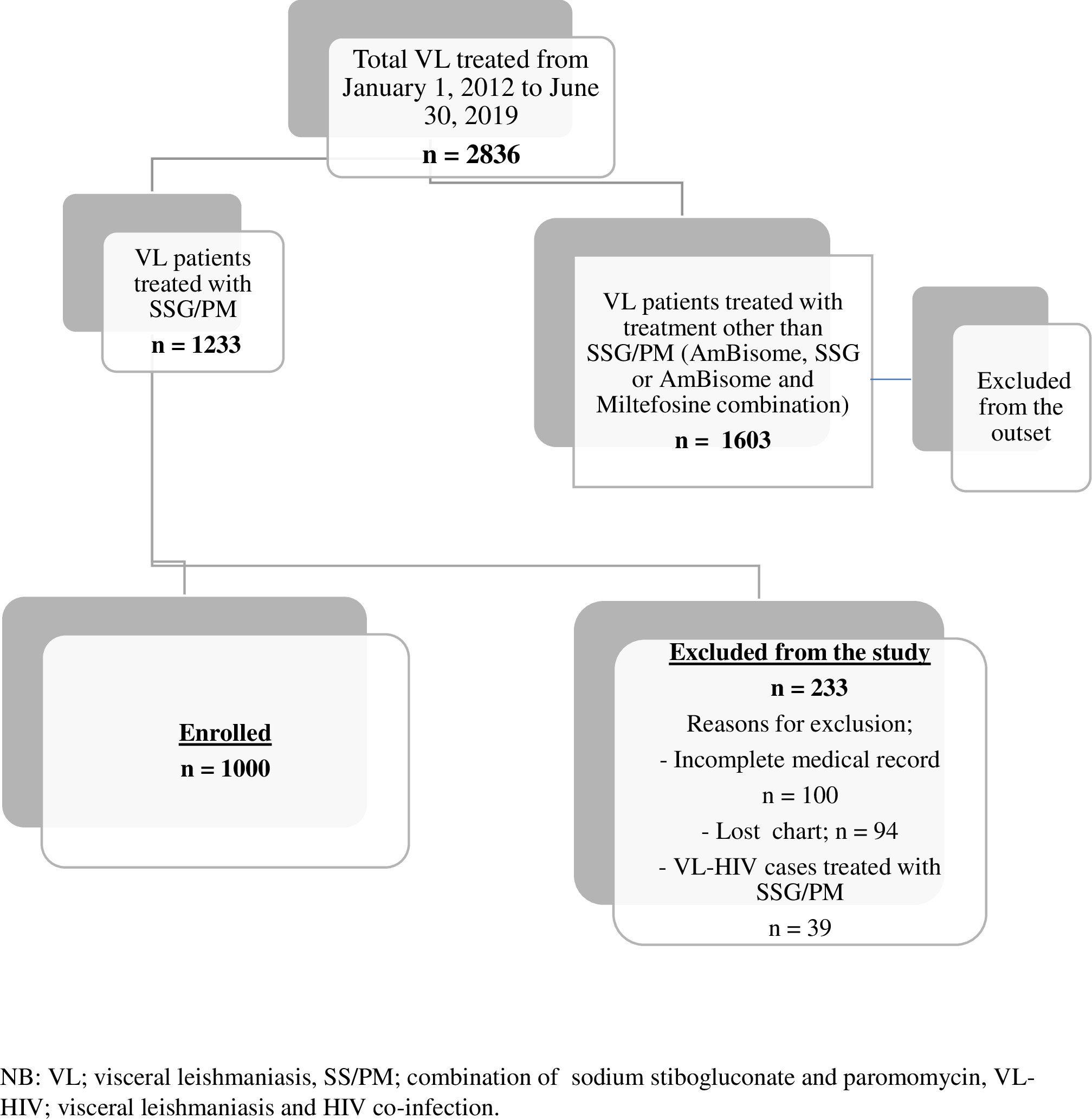

Supplement: S1 Fig — (TIF) [file pntd.0009713.s002.tif]
